# Meal and snack frequency in relation to diet quality in Japanese adults: a cross-sectional study using different definitions of meals and snacks

**DOI:** 10.1017/S0007114520002317

**Published:** 2020-12-14

**Authors:** Kentaro Murakami, Nana Shinozaki, M. Barbara E. Livingstone, Aya Fujiwara, Keiko Asakura, Shizuko Masayasu, Satoshi Sasaki

**Affiliations:** 1 Department of Social and Preventive Epidemiology, School of Public Health, University of Tokyo, Tokyo 113-0033, Japan; 2 Nutrition Innovation Centre for Food and Health (NICHE), School of Biomedical Sciences, Ulster University, Coleraine BT52 1SA, UK; 3 Department of Nutritional Epidemiology and Shokuiku, National Institute of Biomedical Innovation, Health and Nutrition, Tokyo 162-8636, Japan; 4 Department of Environmental and Occupational Health, School of Medicine, Toho University, Tokyo 143-8540, Japan; 5 Ikurien-naka, Ibaraki 311-0105, Japan

**Keywords:** Eating frequency, Meals, Snacks, Diet quality, Japan

## Abstract

Epidemiological evidence on the association between eating frequency and overall diet quality does not represent a consistent picture. This cross-sectional study examined the associations of meal frequency and snack frequency with diet quality, using different definitions of meals and snacks. Based on 4-d weighed dietary record data obtained from 639 Japanese adults aged 20–81 years, all eating occasions were divided into meals or snacks based on either the participant-identified or time-of-day definitions. Diet quality was assessed by the Healthy Eating Index-2015 (HEI-2015) and Nutrient-Rich Food Index 9.3 (NRF9.3). One additional meal per d increased the HEI-2015 total score by 3·6 and 1·3 points based on the participant-identified and time-of-day definitions, respectively. A higher meal frequency was also associated with higher values of some of the HEI-2015 component scores (total vegetables, greens and beans, and total protein foods), irrespective of how meals were defined. Additionally, one additional participant-identified snack per d increased the HEI-2015 total score by 0·7 points. The frequency of participant-identified snacks also showed positive associations with some of the HEI-2015 component scores (total fruits, whole fruits, total vegetables, greens and beans, dairy products, and Na). However, the frequency of time-of-day defined snacks was not associated with the total scores of HEI-2015, although there were some associations for its components. Similar findings were obtained when the NRF9.3 was used. In conclusion, higher meal frequency was consistently associated with higher diet quality, while associations between snack frequency and diet quality varied depending on the definition of snacks.

Epidemiological evidence on the association between total eating frequency (i.e. sum of meal frequency and snack frequency) and overall diet quality does not represent a consistent picture^(1–14)^. These studies should be cautiously interpreted in terms of substantial methodological problems. First, while the assessment of total eating frequency has often relied on a series of non-validated, self-report questions^(1–6)^, only a few studies^(9–14)^ have used comprehensive information on actual dietary habits (using 24-hour dietary recall or dietary record) over multiple days to take into account the relatively large day-to-day variation at the individual level^(15)^. Second, there is no consensus about what constitutes a snack, a meal or an eating occasion, making it complicated to interpret the literature on this topic. Although the majority of researchers have relied on participants’ self-identification of meals, snacks or eating occasions^(1–11,14)^, others have tried to apply more objective criteria such as the time-of-day approach^(12,13,16)^. An accurate distinction between meals and snacks is important given that they are hypothesised to exert different nutritional effects on diet quality^(17)^. Because of the lack of a universally accepted definition of meals and snacks, an understanding of the influence of different meal and snack definitions on the associations between total eating frequency (as well as meal frequency and snack frequency) and diet quality can facilitate the interpretation of the existing literature and help establish consensus on the most appropriate research definition for meals and snacks^(17)^. Third, the associations between eating frequency and diet quality can be confounded by the underreporting of energy intake (EI) particularly by obese or overweight individuals because it can have an influence on both estimates of total eating frequency and diet quality^(11–13)^. Taken together, the diversity of the findings is not at all surprising and merits more robust data analyses to resolve this issue.

The most robust evidence to date about the association between eating frequency and diet quality has focused on Western-type diets^(11–13)^ but not from countries such as Japan. Japanese dietary habits have long attracted interest from other countries, primarily because of their possible contribution to a low prevalence of coronary artery disease and long life expectancy^(18,19)^. In addition to food choice^(20)^, eating patterns probably differ considerably between Japanese and Western populations. For example, the proportion of daily EI consumed as breakfast, lunch, dinner and snacks was, on average, 23, 30, 40 and 8 %, respectively, in Japan^(20)^, while the range of corresponding value was 9–20 % (median 16 %), 16–45 % (25 %), 24–40 % (32 %) and 10–34 % (26 %), respectively, in the USA^(21)^ and ten European countries participating in the European Prospective Investigation into Cancer and Nutrition calibration study^(22)^. Thus, it is conceivable that the associations of eating patterns, including eating frequency, with diet quality may differ between Japan and Western countries.

The aim of this cross-sectional study was to investigate the associations of meal frequency and snack frequency with overall diet quality among Japanese adults, using different definitions of meals and snacks, based on actual intake data derived from a 4-d weighed dietary record.

## Methods

### Data source and analytic sample

The present cross-sectional analysis was based on two independent data collected using the similar procedure but at different time periods, that is, in 2003 and 2013. As details of both surveys have been provided elsewhere^(23–27)^, only a brief description is given here. The former survey (the 2003 survey) was conducted among apparently healthy women and their cohabitating spouses in four geographically diverse areas in Japan: Osaka (urban), Okinawa (urban island), Nagano (rural inland) and Tottori (rural coastal)^(24,25)^. Our recruitment strategy was such that each 10-year age category, namely, 30–39, 40–49, 50–59 and 60–69 years, included eight women for each area (without consideration of age of men), resulting in 256 invited participants. The latter survey (the 2013 survey) was conducted among apparently healthy men and women aged 20–69 years working in welfare facilities (and, in some occasions, their neighbours and acquaintances for age over 60 years) in twenty study areas consisting of twenty-three (out of forty-seven) prefectures^(26,27)^. In the recruitment process, each of the areas included two men and two women from each of five 10-year age groups (20–29, 30–39, 40–49, 50–59 and 60–69 years), resulting in 400 invited participants. Participation of one individual per household was permitted.

In total, 642 participants (*n* 250 in 2003 and 392 in 2013) provided dietary data for the present analysis. After excluding three participants with missing information on the time of eating, which was needed to create eating frequency variables as described below, the present analysis included 639 individuals. None of the sample was a dietitian, had an experience with dietary counselling from a medical doctor or dietitian or had history of hospitalisation for diabetes education.

For both studies, the study purpose and protocol were explained before the study, and written informed consent was obtained from each participant. Use of data from the 2003 survey and the study protocol of the 2013 survey were approved by the University of Tokyo Faculty of Medicine Ethics Committee.

### Dietary assessment

Dietary data were collected by a four-non-consecutive day weighed dietary record during the winter season (February and March) in both surveys^(23,27)^. Each recording period comprised three week days (Monday–Friday) and one weekend day (Saturday or Sunday) in the 2003 survey and three working days and one non-working day in the 2013 survey. Each of recording days was allocated within ~2 weeks by research dietitians. In the latter survey, night shift-working days and days before and after a night shift work were avoided as recording days. Each participant was issued recording sheets and a digital scale (KD-173; Tanita in 2003 and KD-812WH; Tanita in 2013). After receiving written and verbal instructions by a research dietitian, as well as an example of a completed diary sheet, each participant was requested to document and weigh all items eaten or drunk, both in and out of the home, on each of the recording days. On occasions when weighing was problematic (e.g. dining out), they were instructed to document as much information as possible, including the brand name of the food and the consumed portion size (based on typical household measures), as well as the details of leftovers.

The recording sheets for each survey day were submitted directly to the research dietitian after the survey was completed, who then reviewed the forms and, whenever necessary, sought additional information or modification of the record via telephone or in person. All the collected records were then reviewed by research dietitians at each local centre and again at the study centre. As requested in the study protocol, portion sizes estimated using household measures were converted into weights, and individual food items were coded based on the Standard Tables of Food Composition in Japan^(28)^. Estimated intakes of energy and selected nutrients for each individual were calculated based on the intakes of food items and their nutrient contents. Added sugar intake was also calculated based on a recently compiled comprehensive composition database^(29)^.

### Assessment of diet quality

As measures of diet quality, the Healthy Eating Index 2015 (HEI-2015)^(30–32)^ and Nutrient-Rich Food Index 9.3 (NRF9.3)^(33–36)^ were calculated. The HEI-2015 is a 100-point scale to assess compliance with the 2015–2020 Dietary Guidelines for Americans^(37)^, with a higher score indicating a better quality of overall diet. The HEI-2015 consists of nine adequacy components (total fruits, whole fruits, total vegetables, greens and beans, whole grains, dairy products, total protein foods, seafood and plant proteins, and fatty acids as the ratio of the sum of PUFA and MUFA to SFA) and four moderation components (refined grains, Na, added sugars and saturated fats). We calculated the HEI-2015 component and total scores based on energy-adjusted values of overall dietary intake, namely, amount per 4184 kJ (1000 kcal) of energy or percentage of energy, except for fatty acids^(25)^.

The NRF9.3 is a composite measure of the nutrient density of the total diet, calculated as the sum of the percentage of reference daily values for nine qualifying nutrients, namely, protein, dietary fibre, vitamin A, vitamin C, vitamin D, Ca, Fe, K and Mg, minus the sum of the percentage of reference daily values for three disqualifying nutrients, namely, added sugars, saturated fats and Na. Reference daily values were determined for sex and age categories, based on the Dietary Reference Intakes for Japanese, 2015^(38)^, except for added sugars, for which the conditional recommendation advocated by the WHO (i.e. upper limit of 5 % of energy)^(39)^ was used because of the lack of a recommended value for added sugars in Japan, as well as their low intake levels^(29)^. We calculated the NRF9.3 component and total scores based on the overall daily intake of each nutrient for each participant, which was adjusted for EI by the density method and then normalised for the sex- and age-specific Estimated Energy Requirement for a moderate level of physical activity (from the Dietary Reference Intakes for Japanese, 2015^(38)^) and expressed as a percentage of the reference daily value^(25)^. Higher NRF9.3 scores indicated a better quality of the overall diet.

### Definition of meal frequency, snack frequency and total eating frequency

Data from the 4-d dietary record were also used to calculate eating frequency, that is, the number of eating occasions per d. Food intake was documented according to the typical Japanese eating pattern, comprising breakfast, lunch, dinner and snacks, which were prescribed in the diary^(24)^. During the diet recording, participants were asked to record the clock time when a food or beverage was consumed (both start and finish times). In this study, eating occasions were defined as any separate intake occasion with a discrete start clock time and name, except for eating occasions consisting of water only (tap and mineral water), which were excluded^(16)^.

All eating occasions were divided into either meals or snacks with the use of two different published definitions. For the participant-identified definition^(11,13,16)^, eating occasions recorded in the sections of breakfast, lunch and dinner in the food diary were considered meals, while eating occasions recorded in the snack section were considered snacks. We found multiple entries of eating occasions (with different times) into a section of breakfast, lunch or dinner (only ten cases), in which case the first eating occasion was considered a meal, and the following eating occasions were considered snacks. For the time-of-day definition^(12,13)^, meals were defined as eating occasions whose start time was within select time periods of the day (06.00–10.00, 12.00–15.00 and 18.00–21.00 hours), and any eating occasions starting outside of these time periods were considered snacks. These are most widely used definitions of meals and snacks^(16)^.

During data cleaning, it was found that ≥2 different types of eating occasions were recorded within the overlapping time period (243 cases), in which case each of the overlapping eating occasions was combined and counted as a single eating occasion (irrespective of definitions of meals and snacks). For the participant-identified definition, where a participant recorded consuming a meal and a snack within the overlapping period, we considered this eating occasion a meal, unless the participant had already recorded that same meal (breakfast, lunch or dinner) earlier in the day, in which case this eating occasion was considered a snack. For the time-of-day definition, the earliest start time was used to define meals or snacks. For each participant, we calculated meal frequency and snack frequency using the two different definitions, as well as total eating frequency (i.e. sum of meal frequency and snack frequency).

### Assessment of other variables

Body height was measured without shoes to the nearest 0·1 cm. Body weight was measured in light clothing to the nearest 0·1 kg. BMI (kg/m^2^) was calculated as body weight (kg) divided by the square of body height (m), based on which weight status was grouped into three categories of underweight (<18·5 kg/m^2^), normal weight (≥18·5 to <25·0 kg/m^2^) and overweight (≥25·0 kg/m^2^)^(40)^. Misreporting of EI was evaluated on the basis of the ratio of EI:BMR (Goldberg’s cut-off)^(41)^. BMR was estimated according to an equation specifically developed for Japanese on the basis of body height and weight, age and sex^(42,43)^. Assuming a physical activity level for a sedentary lifestyle (i.e. 1·55) for all participants (because of a lack of an objective measure of physical activity), underreporting, plausible reporting and overreporting were defined as having an EI:BMR of <1·02, ≥1·02 to <2·35 and ≥2·35, respectively^(41)^.

### Statistical analysis

All statistical analyses were performed using SAS statistical software (version 9.4, SAS Institute Inc.). All reported *P* values are two-tailed, and *P* values <0·05 were considered statistically significant. For all dietary variables, mean daily values over 4 d were used in the analysis. Descriptive statistics of meal frequency, snack frequency and total eating frequency are presented as means and standard deviations. Differences in meal frequency and snack frequency between the two definitions were examined using paired *t* test. Pearson correlation coefficients among eating frequency variables were calculated. Differences in eating frequency variables and diet quality scores between sex, between survey year and across categories of age (<40, 40–59 and ≥60 years), weight status and dietary reporting status were examined on the basis of independent *t* test or ANOVA followed by Bonferroni’s *post hoc* test. Associations between eating frequency variables and diet quality scores were examined using the general linear model, with adjustment for sex, age group, weight status, dietary reporting status and survey year. In these analyses, eating frequency variables were treated as either a continuous variable or a categorical variable (approximate tertile, except for participant-identified meal frequency, for which only two categories could be made). For the analysis on meal frequency, further adjustment for snack frequency based on the same definition was also made and vice versa. Analyses were repeated after stratified by age (by median), sex or survey year, which provided associations generally similar to those observed in the entire sample (data not shown). The present report, therefore, presents the results for the entire sample.

Power calculations were performed based on the sample size and the standard deviations of eating frequency variables and the total score of HEI-2015 in the present study. We had >80 % power to detect associations as small as 0·55–3·63 point increase of HEI-2015 total score per one increase of meal, snack or total eating frequency per d. Thus, the present study had adequate power to detect a magnitude of associations similar to those reported in a previous study^(13)^.

## Results

The present analysis included 639 Japanese adults with a mean age of 47·1 (sd 13·2) years and a mean BMI of 23·1 (sd 3·4) kg/m^2^. Table 1 shows descriptive statistics of meal, snack and total eating frequency. For both meal frequency and snack frequency, mean values differed significantly between the participant-identified *v*. the time-of-day definitions: 2·92 *v*. 3·56 times/d and 1·76 *v*. 1·12 times/d, respectively. While there was only a weak correlation for meal frequency between the two definitions (Pearson correlation 0·30), the correlation for snack frequency was strong (Pearson correlation 0·74). Both meal frequency and snack frequency were positively correlated with total eating frequency irrespective of definition (Pearson correlation 0·24–0·99).

**Table 1.** Descriptive statistics of meal frequency, snack frequency and total eating frequency in 639 Japanese adults aged 20–81 years (Mean values and standard deviations; median values and 25th and 75th percentiles)

	Mean*	sd	Minimum	Q25	Median	Q75	Maximum	Pearson correlation			
								Meal frequency		Snack frequency	
								Participant identified	Time of day	Participant identified	Time of day
Meal frequency (times/d)											
Participant identified†	2·92	0·20	1·50	3·00	3·00	3·00	3·00	1·00			
Time of day‡	3·56	0·91	0·50	3·00	3·50	4·25	6·75	0·30	1·00		
Snack frequency (times/d)											
Participant identified†	1·76	1·27	0	0·75	1·75	2·50	8·00	0·09	0·77	1·00	
Time of day‡	1·12	0·80	0	0·50	1·00	1·50	6·00	0·05	0·18	0·74	1·00
Total eating frequency (times/d)§	4·68	1·31	1·50	3·75	4·50	5·50	11·00	0·24	0·80	0·99	0·73

Q25, 25th percentile; Q75, 75th percentile.

* For both meal frequency and snack frequency, mean values differed significantly between the two definitions (paired *t* test; *P* < 0·0001).

† For the participant-identified definition, eating occasions recorded in the sections of breakfast, lunch and dinner in the food diary were considered meals, while eating occasions recorded in the snack section were considered snacks. For multiple entries of eating occasions into a section of breakfast, lunch or dinner (ten cases), however, the first eating occasion was considered a meal, and the following eating occasions were considered snacks.

‡ For the time-of-day definition, meals were defined as eating occasions whose start time was within select time periods of the day (06.00–10.00, 12.00–15.00 and 18.00–21.00 hours), and any eating occasions starting outside of these time periods were considered snacks.

§ Sum of meal frequency and snack frequency.

Table 2 shows meal, snack and total eating frequency and diet quality scores according to each category of basic characteristics. Compared with men, women had a higher mean value of all eating frequency variables (except for time-of-day defined meal frequency) and a lower mean value of NRF9.3. Participants aged <40 years and those identified as underreporters had lower meal frequency and diet quality scores than those aged 40–59 and ≥60 years and plausible reporters and overreporters, respectively, irrespective of their definitions. Underreporters also had a lower total eating frequency. Participants in the 2003 survey had a higher frequency of participant-identified meals and higher diet quality scores compared with those in the 2013 survey. There were no differences in any of the eating frequency variables and diet quality scores across weight status categories.

**Table 2.** Meal, snack and total eating frequency and diet quality scores according to each category of basic characteristics in 639 Japanese adults aged 20–81 years (Mean values and standard deviations)

	*n*	Meal frequency (times/d)				Snack frequency (times/d)				Total eating frequency (times/d)‡		HEI-2015§		NRF9.3‖	
		Participant identified*		Time of day†		Participant identified*		Time of day†							
		Mean	sd	Mean	sd	Mean	sd	Mean	sd	Mean	sd	Mean	sd	Mean	sd
Sex															
Male	318	2·90	0·24	3·51	0·95	1·62	1·31	1·02	0·79	4·52	1·34	51·7	7·3	678	104
Female	321	2·94	0·15	3·61	0·86	1·89	1·23	1·21	0·79	4·83	1·26	52·5	7·4	657	107
*P* ¶		0·04		0·13		0·007		0·002		0·003		0·21		0·01	
Age group (years)															
<40	205	2·86^a^	0·28	3·43^a^	0·94	1·71	1·35	1·14	0·84	4·57	1·43	48·2^a^	6·5	615^a^	114
40–59	279	2·94^b^	0·16	3·70^b^	0·90	1·85	1·23	1·09	0·77	4·78	1·23	52·5^b^	6·6	675^b^	92
≥60	155	2·97^b^	0·11	3·49^a,b^	0·84	1·65	1·26	1·13	0·79	4·62	1·27	56·6^c^	6·9	723^c^	84
*P* **		<0·0001		0·003		0·24		0·77		0·17		<0·0001		<0·0001	
Weight status††															
Underweight	35	2·94	0·15	3·71	1·04	2·05	1·42	1·28	0·78	4·99	1·45	50·5	6·2	633	117
Normal weight	445	2·92	0·20	3·58	0·91	1·77	1·24	1·11	0·77	4·69	1·27	52·1	7·2	671	101
Overweight	159	2·91	0·23	3·47	0·86	1·65	1·34	1·09	0·89	4·57	1·38	52·5	7·9	664	116
*P* **		0·82		0·27		0·23		0·45		0·21		0·33		0·10	
Dietary reporting status‡‡															
Underreporting	20	2·43^a^	0·51	2·90^a^	1·26	1·30	1·34	0·83^a^	0·64	3·73^a^	1·49	46·8^a^	9·4	605^a^	150
Plausible reporting	604	2·94^b^	0·16	3·57^b^	0·88	1·77	1·27	1·14^a^	0·80	4·71^b^	1·29	52·2^b^	7·2	671^b^	102
Overreporting	15	3·00^b^	0·00	3·95^b^	1·09	1·67	1·50	0·72^a^	0·67	4·67^a,b^	1·50	54·9^b^	7·9	615^a,b^	159
*P* **		<0·0001		0·001		0·25		0·03		0·004		0·002		0·004	
Survey year															
2003	250	2·96	0·14	3·61	0·80	1·72	1·11	1·07	0·68	4·69	1·13	53·6	6·9	689	96
2013	389	2·89	0·23	3·53	0·97	1·78	1·37	1·14	0·87	4·67	1·41	51·2	7·5	653	110
*P* ¶		<0·0001		0·23		0·62		0·27		0·89		<0·0001		<0·0001	

HEI-2015, Healthy Eating Index-2015; NRF9.3, Nutrient-Rich Food Index 9.3.

* For the participant-identified definition, eating occasions recorded in the sections of breakfast, lunch and dinner in the food diary were considered meals, while eating occasions recorded in the snack section were considered snacks. For multiple entries of eating occasions into a section of breakfast, lunch or dinner (ten cases), however, the first eating occasion was considered a meal, and the following eating occasions were considered snacks.

† For the time-of-day definition, meals were defined as eating occasions whose start time was within select time periods of the day (06.00–10.00, 12.00–15.00 and 18.00–21.00 hours), and any eating occasions starting outside of these time periods were considered snacks.

‡ Sum of meal frequency and snack frequency.

§ A maximum score is 100. A higher score indicates a higher diet quality.

‖ A maximum score is 900. A higher score indicates a higher diet quality.

¶ Based on independent *t* test.

** Based on ANOVA. When the overall *P* from ANOVA was <0·05, Bonferroni’s *post hoc* test was performed; values within each variable with unlike superscript letters are significantly different (*P* < 0·05).

†† Underweight, normal weight and overweight were defined as participants having a BMI (in kg/m^2^) of <18·5, ≥18·5 to <25 and ≥25, respectively.

‡‡ Underreporting, plausible reporting and overreporting were defined as participants having a ratio of reported energy intake to BMR of <1·02, ≥1·02 to <2·35 and ≥2·35, respectively.

The associations between meal frequency and overall diet quality scores are shown in Table 3. In the analysis where meal frequency was treated as a continuous variable, there was a positive association between meal frequency and overall diet quality irrespective of their definitions. For both definitions of meal, a higher meal frequency was associated with a higher value of the HEI-2015 total score, some of the HEI-2015 component scores (including total vegetables, greens and beans, and total protein foods), the NRF9.3 total score and some of the NRF9.3 component scores (including dietary fibre, vitamin C, vitamin D, Ca, K and Mg). For example, one additional meal per d increased the HEI-2015 total score by 3·6 points based on the participant-identified definition and by 1·3 points based on the time-of-day definition. Additionally, the frequency of participant-defined meals was associated with a favourable intake of added sugars (a higher score for HEI-2015 and a lower score for NRF9.3), while the frequency of time-of-day defined meals was associated with higher scores of some other HEI-2015 components, including whole fruits, whole grains, and seafood and plant proteins.

**Table 3.** Associations between meal frequency and diet quality scores in 639 Japanese adults aged 20–81 years* (Mean values and standard deviations; mean values with their standard errors; median values and ranges)

	Mean	sd	Meal frequency (participant identified)†								Meal frequency (time of day)‡									
			Categorical								Categorical									
			C1 (*n* 123)		C2 (*n* 516)		*P*	Continuous			C1 (*n* 220)		C2 (*n* 196)		C3 (*n* 223)		*P*_for trend_	Continuous		
			Mean	se	Mean	se		*β* §	se	*P*	Mean	se	Mean	se	Mean	se		*β* §	se	*P*
Meal frequency (times/d)‖			2·75 (1·5–2·75)		3 (3)						2·75 (0·5–3)		3·5 (3·25–3·75)		4·5 (4–6·75)					
HEI-2015¶	52·1	7·4	50·7	0·6	52·5	0·3	0·01	3·6	1·5	0·01	50·8	0·5	52·6	0·5	53·0	0·4	0·001	1·3	0·3	<0·0001
Total fruits	1·53	1·41	1·40	0·12	1·56	0·06	0·21	0·06	0·28	0·84	1·37	0·09	1·65	0·09	1·58	0·09	0·09	0·11	0·06	0·052
Whole fruits	2·33	1·90	2·02	0·16	2·40	0·07	0·03	0·55	0·37	0·14	2·08	0·11	2·54	0·12	2·39	0·11	0·06	0·18	0·07	0·02
Total vegetables	4·59	0·79	4·41	0·07	4·63	0·03	0·006	0·65	0·17	0·0001	4·47	0·05	4·62	0·05	4·68	0·05	0·005	0·13	0·03	0·0002
Greens and beans	3·43	1·74	3·26	0·15	3·47	0·07	0·23	1·02	0·36	0·005	3·09	0·11	3·69	0·12	3·53	0·11	0·006	0·24	0·07	0·0009
Whole grains	0·63	1·59	0·75	0·15	0·60	0·07	0·38	–0·09	0·36	0·80	0·44	0·11	0·70	0·11	0·74	0·11	0·06	0·17	0·07	0·02
Dairy products	2·08	1·68	1·75	0·15	2·15	0·07	0·02	0·16	0·36	0·65	1·96	0·11	2·06	0·12	2·21	0·11	0·11	0·07	0·07	0·37
Total protein foods	4·75	0·57	4·67	0·05	4·77	0·02	0·11	0·36	0·12	0·004	4·69	0·04	4·80	0·04	4·76	0·04	0·19	0·06	0·03	0·02
Seafood and plant proteins	4·78	0·72	4·74	0·07	4·79	0·03	0·54	0·07	0·16	0·66	4·70	0·05	4·82	0·05	4·82	0·05	0·08	0·09	0·03	0·004
Fatty acids**	6·31	2·58	6·11	0·24	6·36	0·11	0·36	0·30	0·57	0·60	6·51	0·17	5·95	0·18	6·42	0·17	0·75	0·01	0·11	0·94
Refined grains	1·40	2·14	1·46	0·20	1·38	0·09	0·73	–0·23	0·48	0·63	1·31	0·15	1·49	0·15	1·39	0·14	0·72	0·04	0·10	0·67
Sodium	1·96	2·60	2·08	0·23	1·93	0·11	0·59	–0·51	0·56	0·36	1·87	0·17	1·99	0·18	2·03	0·17	0·50	0·09	0·11	0·41
Added sugars	9·42	1·13	9·17	0·10	9·48	0·05	0·009	1·06	0·24	<0·0001	9·43	0·08	9·46	0·08	9·36	0·07	0·52	0·03	0·05	0·52
Saturated fats	8·93	1·65	8·85	0·15	8·95	0·07	0·57	0·20	0·35	0·57	8·93	0·11	8·78	0·11	9·06	0·11	0·38	0·08	0·07	0·28
NRF9.3††	667	106	639	9	674	4	0·0006	118	21	<0·0001	652	7	680	7	671	7	0·054	16·0	4·3	0·0002
Protein	100·0	0·4	99·9	0·0	100·0	0·0	0·16	0·1	0·1	0·20	99·9	0·0	100·0	0·0	100·0	0·0	0·04	0·0	0·0	0·16
Dietary fibre	77·5	16·7	75·0	1·5	78·1	0·7	0·06	8·8	3·5	0·01	75·2	1·1	79·6	1·1	77·9	1·1	0·09	1·7	0·7	0·02
Vitamin A	68·1	21·7	68·3	2·0	68·0	0·9	0·92	8·6	4·7	0·06	66·6	1·4	70·1	1·5	67·8	1·4	0·58	1·1	0·9	0·25
Vitamin C	89·9	16·6	88·7	1·5	90·2	0·7	0·40	11·4	3·6	0·001	87·8	1·1	91·3	1·1	90·8	1·1	0·06	2·0	0·7	0·005
Vitamin D	86·4	22·5	83·3	2·0	87·1	1·0	0·09	14·0	4·8	0·004	83·9	1·5	89·0	1·5	86·5	1·5	0·23	2·0	1·0	0·04
Ca	79·9	18·1	75·3	1·6	81·0	0·8	0·001	10·0	3·8	0·009	76·4	1·2	81·3	1·2	82·2	1·2	0·0006	3·1	0·8	<0·0001
Fe	92·1	13·8	90·6	1·0	92·4	0·5	0·12	4·6	2·5	0·06	91·7	0·8	92·6	0·8	92·0	0·8	0·77	0·6	0·5	0·25
K	92·5	10·7	90·4	0·9	93·0	0·4	0·01	8·4	2·2	0·0002	90·2	0·7	93·8	0·7	93·7	0·7	0·0004	1·9	0·4	<0·0001
Mg	90·4	11·9	87·9	1·0	91·0	0·5	0·008	8·5	2·5	0·0006	88·0	0·8	92·3	0·8	91·1	0·7	0·006	1·8	0·5	0·0003
Added sugars	36·9	52·4	47·5	4·8	34·4	2·2	0·02	–46·4	11·2	<0·0001	34·5	3·5	36·3	3·6	39·9	3·4	0·27	–0·4	2·3	0·88
Saturated fats	20·5	23·3	21·7	2·1	20·2	1·0	0·53	–3·0	5·0	0·55	20·6	1·5	23·0	1·6	18·3	1·5	0·27	–1·3	1·0	0·19
Na	51·9	34·0	51·1	3·0	52·1	1·4	0·76	5·3	7·2	0·46	52·2	2·2	50·5	2·3	52·8	2·2	0·85	–0·1	1·4	0·94

C, category; HEI-2015, Healthy Eating Index-2015; NRF9.3, Nutrient-Rich Food Index 9.3.

* For analyses on the associations between meal frequency and diet quality scores, the meal frequency variable was treated as a categorical variable or a continuous variable based on the general linear model, with adjustment for sex, age group, weight status, dietary reporting status, survey year and snack frequency based on the same definition.

† For the participant-identified definition, eating occasions recorded in the sections of breakfast, lunch and dinner in the food diary were considered meals, while eating occasions recorded in the snack section were considered snacks. For multiple entries of eating occasions into a section of breakfast, lunch or dinner (ten cases), however, the first eating occasion was considered a meal, and the following eating occasions were considered snacks.

‡ For the time-of-day definition, meals were defined as eating occasions whose start time was within select time periods of the day (06.00–10.00, 12.00–15.00 and 18.00–21.00 hours), and any eating occasions starting outside of these time periods were considered snacks.

§ Regression coefficients mean the change of diet quality scores with one additional eating occasion per d.

‖ Values are medians (ranges).

¶ Calculated as the sum of all components scores. A maximum score is 100. A maximum score for each component is as follows: 5 for total fruits, whole fruits, total vegetables, greens and beans, total protein foods and seafood and plant proteins and 10 for whole grains, dairy products, fatty acids, refined grains, Na, added sugars and saturated fats. A higher score indicates a higher diet quality (i.e. a lower intake for refined grains, Na, added sugars and saturated fats components and a higher intake for other components).

** Defined as the ratio of the sum of PUFA and MUFA to SFA.

†† Calculated as the sum of scores for nine nutrients to encourage (i.e. protein, dietary fibre, vitamins A, C and D, Ca, Fe, K and Mg) minus the sum of scores for three nutrients to limit (i.e. added sugars, saturated fats and Na). A maximum score is 900. For each component, a maximum score is 100, except for added sugars, saturated fats and Na components, for which a maximum score is infinite depending on the intake level. A higher score indicates a higher diet quality, except for added sugars, saturated fats and Na components, for which a higher score indicates an unfavourable dietary intake (i.e. higher intakes of added sugars, saturated fats and Na).

On the other hand, the association between snack frequency and overall diet quality differed by the definition of snacks (Table 4). In the analysis where the frequency of participant-identified snacks was treated as a continuous variable, snack frequency showed positive associations with the HEI-2015 total score, some of the HEI-2015 component scores (including total fruits, whole fruits, total vegetables, greens and beans, dairy products, and Na), the NRF9.3 total score and some of the NRF9.3 components (including vitamin C, vitamin D, Ca, K and Mg). For example, one additional participant-identified snack per d increased the HEI-2015 total score by 0·7 points. The frequency of participant-identified snacks was also associated with an unfavourable intake of Na (a lower score for HEI-2015 and a higher score for NRF9.3). However, the frequency of time-of-day snacks was not associated with the total scores of HEI-2015 or NRF9.3, although there were some associations for their component scores.

**Table 4.** Associations between snack frequency and diet quality scores in 639 Japanese adults aged 20–81 years* (Mean values with their standard errors; median values and ranges)

	Snack frequency (participant identified)†										Snack frequency (time of day)‡									
	Categorical										Categorical									
	C1 (*n* 231)		C2 (*n* 176)		C3 (*n* 232)		*P*_for trend_	Continuous			C1 (*n* 190)		C2 (*n* 237)		C3 (*n* 212)		*P*_for trend_	Continuous		
	Mean	se	Mean	se	Mean	se		*β* §	se	*P*	Mean	se	Mean	se	Mean	se		*β* §	se	*P*
Snack frequency (times/d)‖	0·5 (0–1)		1·75 (1·25–2)		2·75 (2·25–8)		–	–	–	–	0·25 (0–0·67)		1 (0·75–1·25)		1·75 (1·5–6)		–	–	–	–
HEI-2015¶	50·7	0·4	52·6	0·5	53·1	0·4	0·0001	0·7	0·2	0·0008	51·6	0·5	51·8	0·4	52·9	0·5	0·07	0·1	0·3	0·66
Total fruits	1·32	0·08	1·66	0·09	1·64	0·08	0·008	0·09	0·04	0·03	1·45	0·09	1·44	0·08	1·70	0·09	0·049	0·06	0·06	0·37
Whole fruits	2·04	0·11	2·56	0·12	2·44	0·11	0·01	0·12	0·05	0·02	2·26	0·12	2·19	0·11	2·55	0·11	0·08	0·08	0·08	0·32
Total vegetables	4·50	0·05	4·52	0·06	4·73	0·05	0·0007	0·08	0·02	0·0005	4·50	0·05	4·56	0·05	4·69	0·05	0·01	0·06	0·04	0·13
Greens and beans	3·13	0·11	3·53	0·12	3·65	0·11	0·0007	0·15	0·05	0·003	3·29	0·12	3·43	0·11	3·55	0·11	0·12	0·09	0·08	0·25
Whole grains	0·45	0·11	0·66	0·12	0·78	0·10	0·03	0·08	0·05	0·10	0·63	0·12	0·70	0·10	0·53	0·11	0·52	–0·03	0·08	0·72
Dairy products	1·86	0·11	2·23	0·12	2·18	0·11	0·04	0·10	0·05	0·04	1·94	0·12	2·02	0·11	2·26	0·11	0·048	0·15	0·08	0·06
Total protein foods	4·71	0·04	4·76	0·04	4·78	0·04	0·18	0·01	0·02	0·51	4·72	0·04	4·76	0·04	4·76	0·04	0·50	–0·02	0·03	0·42
Seafood and plant proteins	4·72	0·05	4·79	0·05	4·83	0·05	0·10	0·04	0·02	0·10	4·77	0·05	4·81	0·05	4·76	0·05	0·88	–0·03	0·04	0·42
Fatty acids**	6·57	0·17	6·12	0·19	6·19	0·17	0·12	–0·13	0·08	0·12	6·45	0·19	6·35	0·17	6·14	0·18	0·23	–0·26	0·13	0·046
Refined grains	1·21	0·14	1·48	0·16	1·52	0·14	0·12	0·09	0·07	0·16	1·29	0·16	1·32	0·14	1·57	0·15	0·19	0·14	0·11	0·21
Na	1·69	0·16	2·09	0·19	2·14	0·16	0·06	0·16	0·08	0·04	1·78	0·18	1·90	0·16	2·20	0·17	0·09	0·21	0·13	0·10
Added sugars	9·61	0·07	9·37	0·08	9·26	0·07	0·0007	–0·12	0·03	0·0007	9·62	0·08	9·40	0·07	9·26	0·08	0·001	–0·22	0·06	<0·0001
Saturated fats	8·93	0·10	8·85	0·12	8·99	0·10	0·72	0·00	0·05	0·98	8·93	0·11	8·98	0·10	8·88	0·11	0·74	–0·08	0·08	0·31
NRF9.3††	658	6	669	7	676	6	0·04	7	3	0·02	658	7	667	6	676	7	0·06	3	5	0·54
Protein	99·9	0·0	100·0	0·0	100·0	0·0	0·06	0·0	0·0	0·12	99·9	0·0	100·0	0·0	100·0	0·0	0·19	0·0	0·0	0·37
Dietary fibre	75·8	1·0	78·0	1·2	78·8	1·0	0·046	0·9	0·5	0·07	76·4	1·1	77·5	1·0	78·4	1·1	0·21	0·4	0·8	0·64
Vitamin A	66·1	1·4	68·9	1·6	69·4	1·4	0·09	1·2	0·7	0·06	65·1	1·5	68·1	1·4	70·7	1·4	0·009	1·8	1·1	0·09
Vitamin C	87·0	1·1	90·2	1·2	92·5	1·0	0·0003	2·4	0·5	<0·0001	86·1	1·2	90·0	1·0	93·2	1·1	<0·0001	3·3	0·8	<0·0001
Vitamin D	84·3	1·4	87·0	1·6	88·0	1·4	0·06	1·7	0·7	0·01	83·9	1·6	87·0	1·4	87·9	1·5	0·07	2·2	1·1	0·049
Ca	75·1	1·1	82·2	1·3	83·0	1·1	<0·0001	2·5	0·5	<0·0001	76·6	1·2	80·5	1·1	82·4	1·2	0·0009	2·4	0·9	0·007
Fe	92·0	0·7	91·4	0·8	92·7	0·7	0·49	0·4	0·3	0·24	91·5	0·8	92·1	0·7	92·6	0·8	0·34	0·4	0·6	0·43
K	90·1	0·7	92·7	0·7	94·8	0·6	<0·0001	1·7	0·3	<0·0001	90·5	0·7	92·5	0·6	94·3	0·7	0·0002	1·8	0·5	0·0004
Mg	88·6	0·7	91·5	0·8	91·4	0·7	0·007	0·9	0·3	0·009	89·7	0·8	90·3	0·7	91·0	0·8	0·24	0·2	0·6	0·68
Added sugars	25·8	3·3	40·5	3·8	45·3	3·3	<0·0001	6·4	1·6	<0·0001	26·1	3·7	37·7	3·3	45·8	3·5	0·0001	11·5	2·6	<0·0001
Saturated fats	20·4	1·5	21·8	1·7	19·7	1·5	0·72	0·0	0·7	0·98	20·7	1·6	19·3	1·4	21·7	1·5	0·61	1·4	1·1	0·20
Na	55·0	2·1	50·2	2·4	50·0	2·1	0·10	–1·7	1·0	0·08	55·4	2·3	53·5	2·1	47·0	2·2	0·009	–3·4	1·6	0·04

C, category; HEI-2015, Healthy Eating Index-2015; NRF9.3, Nutrient-Rich Food Index 9.3.

* For analyses on the associations between snack frequency and diet quality scores, the snack frequency variable was treated as a categorical variable or a continuous variable based on the general linear model, with adjustment for sex, age group, weight status, dietary reporting status, survey year and meal frequency based on the same definition.

† For the participant-identified definition, eating occasions recorded in the sections of breakfast, lunch and dinner in the food diary were considered meals, while eating occasions recorded in the snack section were considered snacks. For multiple entries of eating occasions into a section of breakfast, lunch or dinner (ten cases), however, the first eating occasion was considered a meal, and the following eating occasions were considered snacks.

‡ For the time-of-day definition, meals were defined as eating occasions whose start time was within select time periods of the day (06.00–10.00, 12.00–15.00 and 18.00–21.00 hours), and any eating occasions starting outside of these time periods were considered snacks.

§ Regression coefficients mean the change of diet quality scores with one additional eating occasion per d.

‖ Values are medians (ranges).

¶ Calculated as the sum of all components scores. A maximum score is 100. A maximum score for each component is as follows: 5 for total fruits, whole fruits, total vegetables, greens and beans, total protein foods and seafood and plant proteins and 10 for whole grains, dairy products, fatty acids, refined grains, Na, added sugars and saturated fats. A higher score indicates a higher diet quality (i.e. a lower intake for refined grains, Na, added sugars and saturated fats components and a higher intake for other components).

** Defined as the ratio of the sum of PUFA and MUFA acids to SFA.

†† Calculated as the sum of scores for nine nutrients to encourage (i.e. protein, dietary fibre, vitamins A, C and D, Ca, Fe, K and Mg) minus the sum of scores for three nutrients to limit (i.e. added sugars, saturated fats and Na). A maximum score is 900. For each component, a maximum score is 100, except for added sugars, saturated fats and Na components, for which a maximum score is infinite depending on the intake level. A higher score indicates a higher diet quality, except for added sugars, saturated fats and Na components, for which a higher score indicates an unfavourable dietary intake (i.e. higher intakes of added sugars, saturated fats and Na).

Consistent with these findings, total eating frequency (sum of meal frequency and snack frequency) was positively associated with overall diet quality (online Supplementary Table S1). For example, one additional eating occasion per d increased the HEI-2015 total score by 0·8 points. When the analyses were repeated treating each of eating frequency variables as a categorical variable, generally similar results were obtained (Table 3 for meal frequency, Table 4 for snack frequency and online Supplementary Table S1 for total eating frequency).

## Discussion

To our knowledge, this is the first study to examine associations of different measures of meal frequency and snack frequency with diet quality in Japanese adults. After adjustment for potential confounding factors, we found that a higher meal frequency was associated with a higher quality of overall diet as assessed by the HEI-2015 and NRF9.3. This was not dependent on the definition of meals because the results were similar for both the participant-identified and time-of-day approaches. On the other hand, associations between snack frequency and diet quality varied depending on the definition of snacks. The frequency of participant-identified snacks, but not that of time-of-day defined ones, showed positive associations with overall diet quality. Thus, the present results suggest the different effects of meal frequency and snack frequency on diet quality as well as the importance of definitions of meals and snacks applied.

There have been only a few attempts to separately investigate the effects of meal frequency and snack frequency on diet quality. A study in a representative sample of American adults^(13)^ found positive associations of both meal frequency and snack frequency with diet quality assessed by the Healthy Eating Index-2010 irrespective of the definitions of meals and snacks, that is, based on time of day and based on self-report (as well as based on EI contribution, which was not used in the present study mainly because of a lack of sufficient information for determining an appropriate cut-off point in Japanese). An analysis based on the UK National Diet and Nutrition Survey showed that, using the time-of-day and EI contribution approaches, a higher snack frequency was consistently associated with lower diet quality assessed by the Healthy Diet Indicator and Mediterranean Diet Score, while associations for meal frequency varied depending on sex and on the definition of meals^(12)^. In a representative sample of Australian adults, the frequency of participant-identified meals, but not snacks, was positively associated with overall diet quality as assessed with the use of the 2013 Australian Dietary Guidelines Index^(11)^. Using the participant-identified and time-of-day approaches, the present study found consistent associations between meal frequency and diet quality, while the associations for snack frequency varied depending on its definition, which broadly concurs with the Australian study. The diversity of the findings may be, at least partly, due to the diversity of the characteristics and lifestyles of the populations examined, dietary assessment methods and diet quality measures applied, definitions of meals and snacks and potential confounding factors considered.

The positive association between meal frequency and diet quality we observed here may be due to the food profiles of Japanese meals. According to a secondary analysis based on the National Health and Nutrition Survey, meals (breakfast, lunch and dinner) were characterised by a high intake of a variety of foods such as vegetables, fruits, dairy products, fish and pulses^(20)^. Additionally, these meals typically contribute to >90 % of total EI^(20)^. The present findings thus suggest that meals are an important determinant of diet quality in Japanese and should be interpreted as evidence that meal skipping may result in lowering diet quality (rather than increasing meal frequency as an effective strategy for improving diet quality). On the other hand, the associations between snack frequency and diet quality varied depending on the definition of snacks, with the frequency of participant-identified snacks, but not that of time-of-day defined ones, showing positive associations. This is most probably a reflection of the heterogeneous nature of snacking behaviours in terms of nutritional and food profiles. In a Japanese study, for example, snacks were characterised by a high intake of not only foods such as confectioneries and soft drinks but also foods such as dairy products and fruit^(44)^. Thus, snack behaviours may represent some opportunity for improving overall diet quality, although this should not be overstressed considering that snacks contribute only about 8 % of total EI in Japan^(20)^.

Reaching a consensus on the most appropriate definition of meals and snacks is likely to remain elusive. While the participant-identified meal frequency and snack frequency have been most widely used, these are subject to inconsistencies due to differences in individual perceptions of meals and snacks and would not necessarily have reduced bias^(45)^. The meal frequency and snack frequency based on time of day may also be problematic because eating patterns vary according to lifestyle as well as the cultural environment^(45)^. In the present study, 90 % of participant-identified meals were categorised into time-of-day defined meals (6703 of 7459). This may also help to explain the consistent associations between meal frequency and diet quality and suggest that the use of these two different definitions are interchangeable in the context of the Japanese diet. Conversely, only 53 % of participant-identified snacks were categorised into time-of-day defined snacks (2390 of 4485) and reflected in inconsistent associations between snack frequency and diet quality. While it is reasonable to assume that people may consider their dessert as a snack, this dessert may be mainly composed of foods potentially related to higher diet quality (e.g. dairy products and fruits). Alternatively, the dessert may be mainly composed of foods potentially related to lower diet quality (e.g. confectioneries). The nutritional quality of snacks may thus be largely dependent on the choice of foods, which may also be associated with the time of day of eating. Thus, the choice of snack definition may have substantial impact on the outcomes. In any case, similar research using different definitions of meals and snacks needs to be accumulated before reaching a consensus on what is the most meaningful way to define meals and snacks in the context of the Japanese diet.

The strength of this study is the use of two different published definitions of meal frequency and snack frequency based on detailed dietary information obtained from a 4-d weighed dietary record. However, there are also several limitations. First, although sampling was conducted to consider regional differences in dietary habits, the present population is not a nationally representative sample of general Japanese, but rather volunteers. In particular, our participants may be biased towards greater health consciousness. Nevertheless, the mean values and standard deviations of the HEI-2015 in the present population were comparable with those reported from the 2012 Japanese National Health and Nutrition Survey (51·3 (9·0) for men and 52·9 (9·2) for women; information not available for eating frequency variables and NRF9.3)^(46)^. Conversely, mean snack frequency (based on the participant-identified definition) in this population was lower than that reported from Western countries (2·3–4·1 times per d)^(21,22)^. Further research in a more representative sample is needed.

Second, all self-reported dietary assessment methods are subject to both random and systematic errors^(47)^, and the nature and extent of the measurement error of self-report-based information on eating patterns, including eating frequency, are largely unknown^(21)^. The present results should therefore be interpreted with caution in this respect. To minimise the influence of measurement error in dietary variables, we included dietary reporting status as a covariate as well as the use of energy-adjusted values of all the diet quality measures^(48)^.

Third, diet quality was assessed by the HEI-2015 and NRF9.3 in this Japanese study, even though both scores were primarily developed for assessing the US diet and not optimal for assessing the overall quality of Japanese diet, which, nevertheless, remain the most comprehensive indices available^(48)^. The use of other diet quality scores, such as the Dietary Inflammatory Index^(49)^, which is not culture bound, would be of interest in future studies. However, in our recent systematic review of Japanese studies which resulted in the identification of dietary patterns using principal component analysis, we found that those food groups which contributed to dietary patterns termed healthy (fruits, vegetables, potatoes, mushrooms, seaweeds and pulses) are at least partly similar to those often observed in Western countries (fruits, vegetables including mushrooms, poultry, fish, low-fat dairy products, legumes and whole grains)^(50)^. It should also be stressed that our recent analysis supports the efficacy of these measures in assessing the overall diet quality of Japanese: a higher total score in the HEI-2015, and NRF9.3 was associated with favourable patterns of overall diet, including higher intakes of dietary fibre and key vitamins and minerals and lower intakes of saturated fats, added sugars and Na^(48)^. In addition, although we adjusted for a variety of potential confounding variables, residual confounding could not be ruled out. Finally, in view of the multiple analyses, it is possible that some of the significant findings in the present study occurred by chance.

To conclude, in this cross-sectional study in Japanese adults, after taking into account potential confounding factors, a higher frequency of both participant-identified and time-of-day defined meals was associated with a higher quality of overall diet. Conversely, associations for snack frequency were dependent on the definition of snacks, as the frequency of participant-identified snacks, but not that of time-of-day defined ones, showed positive associations with overall diet quality. The present results suggest the different effects of meal frequency and snack frequency on diet quality as well as the importance of definitions of meals and snacks applied. Further research on how the nutritional and food profiles of meals and snacks are associated with overall diet quality is warranted to help inform the development of strategies and messages that encourage healthy eating patterns.

## Supplementary material

For supplementary material accompanying this paper visit https://doi.org/10.1017/S0007114520002317. click here to view supplementary material
