# Supplementary material for: Meal and snack frequency in relation to diet quality in Japanese adults: a cross-sectional study using different definitions of meals and snacks
Source: Br J Nutr. 2020 Jun 29;124(11):1219–28. doi: 10.1017/S0007114520002317 (PMC7653514; doi:10.1017/S0007114520002317)
Supplement: Supplementary file 1 [file S0007114520002317sup001.docx]

Supplementary Table S1. Associations between total eating frequency and diet quality scores in 639 Japanese adults aged 20-81 years*

|  |  |  | Categorical | | |  |  |  |  |  |
| --- | --- | --- | --- | --- | --- | --- | --- | --- | --- | --- |
|  | C1 (*n* 231) | | C2 (*n* 176) | | C3 (*n* 232) | | *P* for | Continuous | | |
|  | Mean | SE | Mean | SE | Mean | SE | trend | β† | SE | *P* |
| Total eating frequency (times/d)‡ | 0.5 (0-1) | | 1.75 (1.25-2) | | 2.75 (2.25-8) | | --- | --- | --- | --- |
| HEI-2015§ | 50.6 | 0.5 | 52.2 | 0.4 | 53.3 | 0.4 | <0.0001 | 0.8 | 0.2 | 0.0001 |
| Total fruits | 1.40 | 0.09 | 1.53 | 0.08 | 1.64 | 0.08 | 0.053 | 0.09 | 0.04 | 0.02 |
| Whole fruits | 2.10 | 0.12 | 2.40 | 0.11 | 2.46 | 0.11 | 0.04 | 0.13 | 0.05 | 0.008 |
| Total vegetables | 4.43 | 0.06 | 4.57 | 0.05 | 4.75 | 0.05 | <0.0001 | 0.10 | 0.02 | <0.0001 |
| Greens and beans | 3.04 | 0.12 | 3.51 | 0.11 | 3.66 | 0.11 | 0.0002 | 0.18 | 0.05 | 0.0004 |
| Whole grains | 0.46 | 0.12 | 0.60 | 0.11 | 0.79 | 0.11 | 0.04 | 0.08 | 0.05 | 0.11 |
| Dairy | 1.87 | 0.12 | 2.14 | 0.11 | 2.19 | 0.11 | 0.06 | 0.11 | 0.05 | 0.04 |
| Total protein foods | 4.66 | 0.04 | 4.79 | 0.04 | 4.78 | 0.04 | 0.050 | 0.02 | 0.02 | 0.22 |
| Seafood and plant proteins | 4.71 | 0.05 | 4.80 | 0.05 | 4.82 | 0.05 | 0.13 | 0.04 | 0.02 | 0.09 |
| Fatty acids\|\| | 6.62 | 0.19 | 6.15 | 0.17 | 6.22 | 0.17 | 0.14 | -0.11 | 0.08 | 0.15 |
| Refined grains | 1.07 | 0.16 | 1.53 | 0.14 | 1.54 | 0.14 | 0.04 | 0.08 | 0.07 | 0.20 |
| Sodium | 1.72 | 0.18 | 2.00 | 0.16 | 2.12 | 0.17 | 0.11 | 0.14 | 0.08 | 0.06 |
| Added sugars | 9.60 | 0.08 | 9.37 | 0.07 | 9.31 | 0.07 | 0.01 | -0.08 | 0.03 | 0.02 |
| Saturated fats | 8.97 | 0.12 | 8.85 | 0.10 | 8.98 | 0.11 | 0.87 | 0.00 | 0.05 | 0.93 |
| NRF9.3¶ | 653 | 7 | 669 | 6 | 679 | 6 | 0.008 | 10 | 3 | 0.0006 |
| Protein | 99.9 | 0.0 | 100.0 | 0.0 | 100.0 | 0.0 | 0.02 | 0.0 | 0.0 | 0.07 |
| Dietary fiber | 75.7 | 1.2 | 77.7 | 1.0 | 78.8 | 1.1 | 0.06 | 1.1 | 0.5 | 0.02 |
| Vitamin A | 65.6 | 1.5 | 68.9 | 1.4 | 69.3 | 1.4 | 0.09 | 1.4 | 0.6 | 0.03 |
| Vitamin C | 86.4 | 1.2 | 89.9 | 1.1 | 92.8 | 1.1 | <0.0001 | 2.6 | 0.5 | <0.0001 |
| Vitamin D | 82.7 | 1.6 | 87.3 | 1.4 | 88.6 | 1.4 | 0.009 | 2.1 | 0.7 | 0.002 |
| Calcium | 74.7 | 1.3 | 80.9 | 1.1 | 83.3 | 1.1 | <0.0001 | 2.8 | 0.5 | <0.0001 |
| Iron | 91.3 | 0.8 | 92.1 | 0.7 | 92.7 | 0.7 | 0.22 | 0.5 | 0.3 | 0.12 |
| Potassium | 89.7 | 0.7 | 92.5 | 0.7 | 94.9 | 0.7 | <0.0001 | 1.9 | 0.3 | <0.0001 |
| Magnesium | 88.4 | 0.8 | 91.0 | 0.7 | 91.4 | 0.7 | 0.009 | 1.1 | 0.3 | 0.001 |
| Added sugars | 26.5 | 3.7 | 39.3 | 3.4 | 43.4 | 3.4 | 0.001 | 5.0 | 1.6 | 0.002 |
| Saturated fats | 20.2 | 1.6 | 21.6 | 1.5 | 19.7 | 1.5 | 0.77 | -0.1 | 0.7 | 0.93 |
| Sodium | 55.2 | 2.4 | 50.8 | 2.1 | 50.2 | 2.2 | 0.13 | -1.6 | 1.0 | 0.12 |

C, category; HEI-2015, Healthy Eating Index-2015; NRF9.3, Nutrient-Rich Food Index 9.3.

* For analyses on the associations between total eating frequency and diet quality scores, the total eating frequency variable was treated as a categorical variable or a continuous variable based on the general linear model, with adjustment for sex, age group, weight status, dietary reporting status, and survey year. Total eating frequency was calculated as the sum of meal frequency and snack frequency.

† Regression coefficients mean the change of diet quality scores with one additional eating occasion per day.

‡ Values are medians (ranges).

§ Calculated as the sum of all components scores. A maximum score is 100. A maximum score for each component is as follows: 5 for total fruits, whole fruits, total vegetables, greens and beans, total protein foods, and seafood and plant proteins and 10 for whole grains, dairy, fatty acids, refined grains, sodium, added sugars, and saturated fats. A higher score indicates a higher diet quality (i.e., a lower intake for refined grains, sodium, added sugars, and saturated fats components and a higher intake for other components).

|| Defined as the ratio of the sum of polyunsaturated and monounsaturated fatty acids to saturated fatty acids.

¶ Calculated as the sum of scores for nine nutrients to encourage (i.e., protein, dietary fiber, vitamins A, C and D, calcium, iron, potassium, and magnesium) minus the sum of scores for three nutrients to limit (i.e., added sugars, saturated fats, and sodium). A maximum score is 900. For each component, a maximum score is 100, except for added sugars, saturated fats, and sodium components, for which a maximum score is infinite depending on the intake level. A higher score indicates a higher diet quality, except for added sugars, saturated fats, and sodium components, for which a higher score indicates an unfavorable dietary intake (i.e., higher intakes of added sugars, saturated fats, and sodium).
